# Is intuitive eating a privileged approach? Cross-sectional and longitudinal associations between food insecurity and intuitive eating

**DOI:** 10.1017/S1368980023000460

**Published:** 2023-07

**Authors:** C Blair Burnette, Vivienne M Hazzard, Nicole Larson, Samantha L Hahn, Marla E Eisenberg, Dianne Neumark-Sztainer

**Affiliations:** 1 Division of Epidemiology and Community Health, School of Public Health, University of Minnesota, Minneapolis, MN, USA; 2 Department of Psychiatry and Behavioral Sciences, University of Minnesota Medical School, Minneapolis, MN, USA; 3 Central Michigan University College of Medicine, Mt. Pleasant, MI, USA; 4 Division of General Pediatrics and Adolescent Health, Department of Pediatrics, University of Minnesota Medical School, Minneapolis, MN, USA

**Keywords:** Intuitive eating, Food insecurity, Adolescence, Emerging adulthood, Health equity

## Abstract

**Objective:**

To examine: (1) cross-sectional and longitudinal associations between measures of food insecurity (FI; household status and youth-reported) and intuitive eating (IE) from adolescence to emerging adulthood; and (2) the association between FI persistence and IE in emerging adulthood.

**Design:**

Longitudinal population-based study. Young people reported IE and FI (two items from the US Household Food Security Module) in adolescence and emerging adulthood. Parents provided data on household FI via the six-item US Household Food Security Module in adolescence.

**Setting:**

Adolescents (*M*_age_ = 14·3 ± 2 years) and their parents, recruited from Minneapolis/St. Paul public schools in 2009–2010 and again in 2017–2018 as emerging adults (*M*_age_ = 22·1 ± 2 years).

**Participants:**

The analytic sample (*n* 1372; 53·1 % female, 46·9 % male) was diverse across race/ethnicity (19·8 % Asian, 28·5 % Black, 16·6 % Latinx, 14·7 % Multiracial/Other and 19·9 % White) and socio-economic status (58·6 % low/lower middle, 16·8 % middle and 21·0 % upper middle/high).

**Results:**

In cross-sectional analyses, youth-reported FI was associated with lower IE during adolescence (*P* = 0·02) and emerging adulthood (*P* < 0·001). Longitudinally, household FI, but not adolescent experience of FI, was associated with lower IE in emerging adulthood (*P* = 0·01). Those who remained food-insecure (*P* = 0·05) or became food-insecure (*P* = 0·02) had lower IE in emerging adulthood than those remaining food-secure. All effect sizes were small.

**Conclusions:**

Results suggest FI may exert immediate and potentially lasting impacts on IE. As evidence suggests IE is an adaptive approach conferring benefits beyond eating, it would be valuable for interventions to address social and structural barriers that could impede IE.

Food insecurity (FI; i.e. lack of consistent, dependable access to sufficient food for an active, healthy life) affected 10·5 % of households in the USA in 2020, and almost 4 % of households experienced *very low* food security, meaning at least one family member’s food intake was reduced due to limited resources for food^(1)^. FI is an important health equity issue in the USA that disproportionately affects certain groups. For instance, relative to the national average (10·5 %), FI prevalence was significantly higher in 2020 among non-Hispanic Black households (21·7 %), Hispanic households (17·2 %) and households with children (14·8 %), but significantly *lower* among non-Hispanic White households^(1)^. These disparities have widened since the onset of the COVID-19 pandemic^(1)^, which is of serious concern given that FI is a primary social determinant of health that is associated with chronic disease and worsened mental health^(2,3)^.

Growing evidence documents associations between FI and disordered eating behaviours^(4)^. Cross-sectionally, FI has been linked to engagement in extreme weight-control behaviours, binge eating and a greater likelihood of screening positive for an eating disorder among adolescents and young adults^(5,6)^. Recent longitudinal evidence also found that FI in adolescence predicted later binge eating^(5)^. Fluctuations in food availability are thought to contribute to these associations, as individuals must modify their intake in response to alternating periods of food scarcity and availability at home^(4)^. This hypothesis is supported by research into the neurobiological, psychological and behavioural effects of food deprivation. For instance, there is evidence that dietary restraint (i.e. attempting to restrict food intake) is associated with greater reward responsivity and reward-related learning, such that foods may become more exciting when available again^(7)^. This association does not appear to be limited to those engaging in restraint driven by weight or shape concerns, as women with a history of poverty-associated food deprivation described the feelings of pleasure and anticipation that accompanied food availability in qualitative interviews^(8)^. Caloric restriction can increase attentional bias to food cues, particularly for foods that are highly palatable and calorie-dense^(9)^. This effect might be further compounded for those experiencing FI, as hyper-palatable and calorie-dense foods are often more readily accessible and affordable^(10)^. When food is scarce or intermittently available, seeking higher calorie foods or eating large quantities might be evolutionarily adaptive but could also drive the link between FI and binge eating^(4,5)^. FI is also associated with higher BMI^(11)^, which could partially explain the associations between FI and extreme weight-control behaviours. As westernised cultures emphasise leanness while devaluing and stigmatising higher weight^(12)^, those with a history of FI and high BMI might be more likely to use extreme weight-control behaviours to attempt weight loss.

The theory that dietary restraint drives disordered behaviours such as binge eating also underlies the rationale for an eating approach known as intuitive eating (IE). IE is a multidimensional framework that emphasises eating according to hunger and satiety cues^(13)^. According to IE, letting go of dieting is a necessary first step because non-medically necessary food rules can disrupt one’s attunement to their internal cues and increase the risk for eating in the absence of hunger^(13)^. There is growing support for IE as an adaptive eating style that confers benefits beyond eating^(14)^. For example, there is cross-sectional evidence of IE’s associations with lower disordered eating, body dissatisfaction, and depressive symptoms, and higher emotional functioning and fruit and vegetable intake^(14,15)^. Though longitudinal and intervention research is still nascent, preliminary evidence is encouraging. For example, longitudinal evidence suggests that IE predicts better psychological health and less disordered eating in community samples^(16)^, and that women with gestational diabetes who had higher IE during the perinatal period displayed improved blood glucose control postpartum relative to women with lower IE^(17)^. Further, IE interventions have resulted in decreases in weight-bias internalisation and disordered eating and improvements in body image and quality of life, though most interventions have been conducted with White women, limiting the generalisability of findings^(18,19)^.

Despite these benefits, there is mainstream criticism that IE might be a privileged approach that is simply less accessible for those at lower socio-economic status (SES)^(20,21)^. The argument is that many of IE’s principles conflict with the options available to those with limited resources for food. First, those who lack dependable access to food may have to eat according to food availability *v*. hunger cues, which may increase the likelihood of eating past satiety when food is present^(22)^. Additionally, there is evidence that among households experiencing FI, the head of the household is more likely to work multiple jobs and have varied, changing schedules, which can disrupt both personal and family feeding routines^(23)^. IE also emphasises eating satisfying foods that feel good in the body, such as those that enhance energy levels and do not cause stomach upset^(13)^. However, those at lower SES are more likely to live in neighbourhoods without access to large grocery stores or fresh produce^(24)^, thereby limiting the types of food available. Moreover, fresh produce is less affordable than packaged foods and requires both greater time and financial resources to prepare^(25)^. Finally, FI is associated with heightened emotional stress, depression and anxiety, which can disrupt attunement to internal cues and increase the risk for maladaptive eating behaviours^(3,26)^. Therefore, in the acute context of FI, individuals may not be able to immediately honour their hunger cues, nor eat a variety of nutritious foods that support feelings of satisfaction and satiety. However, given the aforementioned biobehavioural insights into the so-called ‘paradoxical’ effects of food deprivation^(9,11)^, it also seems plausible that FI could exert lasting impacts on one’s ability to eat intuitively.

Although there are reasons to believe IE might be more viable for those with economic privilege, there is no known longitudinal research on differences in IE by food security status. A recent cross-sectional paper found that adults (*M*_age_ = 47·3) experiencing FI had significantly lower IE than those who were food-secure^(27)^. Therefore, the purpose of the current study was to build on this work by examining: (1) cross-sectional associations between FI and IE in both adolescence and emerging adulthood; (2) longitudinal associations between FI in adolescence and IE in emerging adulthood; and (3) whether the persistence of FI from adolescence to emerging adulthood is associated with IE in a younger population-based sample that is racially, ethnically and socio-economically diverse. Because data suggest children and parents experience FI differently and their reports do not always agree^(28)^, we included measures of household (i.e. parent-reported) and youth experience of FI in adolescence. We hypothesised that FI would be related to lower IE in both cross-sectional and longitudinal analyses. We expected that the young person’s experience of FI would display stronger associations with IE than the household measure, given evidence that adults in the household often try to shield their children from FI’s effects^(29)^. Further, we expected that young people who remained food-insecure into emerging adulthood would demonstrate lower IE than their peers without current or previous FI. Results will help highlight whether FI might impede access to an adaptive and health-promoting eating style, which can inform future work aimed at reducing structural barriers to nutritional well-being and increasing health equity.

## Method

### Study design and population

EAT 2010–2018 (Eating and Activity over Time) is a population-based, longitudinal study of eating, activity, and weight-related attitudes, behaviours, and associated sociocultural factors from adolescence to adulthood^(30)^. Middle- and high-school students enrolled at twenty urban public schools in the Minneapolis-St. Paul, Minnesota metropolitan area were recruited for the study. Participants completed surveys twice: at baseline during the 2009–2010 academic year (mean age = 14·4 ± 2·0 years) and at follow-up in 2017–2018 (EAT 2018; mean age = 22·0 ± 2·0 years). Of the original sample (*n* 2793), 65·8 % (*n* 1568) completed surveys at both time points; only participants who responded at both EAT 2010 and 2018 are included in the current analysis. Attrition did not occur at random; individuals identifying as female and White at higher SES were more likely to respond. Thus, we used inverse probability weighting to minimise response bias and allow extrapolation back to the original EAT 2010 sample^(31)^. Inverse probability weighting weights were derived based on characteristics reported in 2010, including sociodemographic information, past-year dieting frequency and weight status. Up to two parents/guardians of each participant were invited to participate in the coordinated Project F-EAT 2010 study^(32)^, and at least one parent/guardian responded for 85·3 % of the adolescent sample; adolescent and parent data were linked by anonymous record numbers.

The analytic sample included 1372 participants (53·1 % female, 46·9 % male) who provided IE and food security data at both time points (EAT 2010 and 2018) and had a parent/guardian provide household food security data in adolescence. Relative to national data^(33)^, the weighted sample was diverse across race and ethnicity (19·8 % Asian, 28·5 % Black, 16·6 % Latinx, 14·7 % Multiracial/Other and 19·9 % White). In adolescence, 58·6 % of the sample were at low/lower middle SES, 16·8 % middle and 21·1 % upper middle/high.

### EAT survey development

The EAT 2010 survey included 235 self-report items assessing a range of factors of potential relevance to weight-related health among adolescents. Development of this survey was guided by a review of previous Project EAT surveys to identify key items, a theoretical framework which integrates an ecological perspective with Social Cognitive Theory, multidisciplinary expert review and pilot testing with adolescents^(34–36)^. Test–retest reliability of measures was examined using data from a subgroup of 129 adolescents who completed the EAT 2010 survey twice within one week. Similarly, measures included on the Project F-EAT written survey and telephone interview were reviewed by a panel of content-area experts and multicultural research staff to address cultural sensitivity and were pilot tested with parents of adolescents. Details of the EAT 2010 and F-EAT survey development process and survey psychometrics have been previously published^(37)^. Key items from the EAT 2010 survey were retained at EAT 2018 to facilitate longitudinal analyses. EAT 2010 survey items were also modified for EAT 2018, when appropriate, to reflect secular trends and the developmental transition from adolescence to emerging adulthood. Focus groups were held with twenty-nine young people to evaluate the 2018 survey; once finalised, a subgroup of EAT 2018 participants (*n* 112) completed the survey twice within 3 weeks to assess test–retest reliability.

## Measures

### Food insecurity

#### Household food insecurity

In 2010, the six-item US Household Food Security Survey Module (modified for self-report) was administered to parents/guardians of adolescent participants to assess food security over the prior 12 months^(38,39)^. The six-item survey module has demonstrated the ability to correctly classify the food security status of 97·7 % of households compared with the full, eighteen-item scale^(38)^. Affirmative responses were summed; ≥ 2 affirmative responses indicates low food security and ≥ 5 indicates very low food security^(38)^. We dichotomised the variable, coding households with < 2 affirmative responses as food-secure and those with > 2 as food-insecure; agreement of the dichotomised variable was 90 %.

#### Food insecurity experience of young people

At each time point, participants responded to two items from the US Household Food Security Module^(39)^. At EAT 2010, adolescents who experienced any hunger and any food inadequacy in the home in the prior 12 months were considered food-insecure (test–retest agreement = 96 %). At EAT 2018, emerging adults were considered food-insecure if they had experienced any hunger and ever eaten less than they should because they did not have enough money for food in the prior 12 months.

#### Food insecurity persistence

Persistence of food security was based on young peoples’ experiences of FI in adolescence and emerging adulthood. We created four categories: (1) remained food-secure (coded as food-secure in both adolescence and emerging adulthood); (2) became food-secure (coded as food-insecure in adolescence but food-secure in emerging adulthood); (3) became food-insecure (coded as food-secure in adolescence but food-insecure in emerging adulthood); (4) remained food-insecure (coded as food-insecure in both adolescence and emerging adulthood).

### Intuitive eating

At both time points, IE was assessed via the following three items adapted from the Intuitive Eating Scale (IES)^(40)^: ‘I stop eating when I feel full’, ‘I eat everything that is on my plate, even if I’m not that hungry’ (reverse-coded) and ‘I trust my body to tell me how much to eat’. Items were rated on a four-point scale, from 1 = *Hardly ever* to 4 = *Almost always,* and averaged to derive an overall IE score, with higher scores reflecting greater IE (McDonald’s ω = 0·50 (2010); 0·56 (2018); test–retest = 0·57 at follow-up).

### Demographics

Participants self-reported their sex, age, race and ethnicity at EAT 2010. Responses options for race/ethnicity were (1) White, (2) Black or African–American, (3) Hispanic or Latino, (4) Asian–American, (5) Hawaiian or Pacific Islander, (6) American Indian or Native American and (7) Multiracial or other race, with participants able to check all that applied (test–retest agreement = 92 %). Because few participants identified as Hawaiian/Pacific Islander and American Indian/Native American, those youth were categorised as Multiracial/Other. A classification and regression tree-based algorithm determined SES based on the following socio-economic indicators: parental educational attainment, employment status and public assistance receipt^(41)^.

### Data analysis

Analyses incorporated inverse probability weighting to account for attrition over time and were conducted in R v4.1.0^(42)^. Descriptive statistics were calculated. Paired-samples *t* tests evaluated whether IE scores changed significantly from adolescence to emerging adulthood. We evaluated statistical significance at *P* < 0·05.

To evaluate cross-sectional and longitudinal associations of FI and IE, we conducted linear regression models with FI status as the independent variable and IE as the dependent variable. Unadjusted and adjusted models were conducted, with adjusted models including age, sex, race/ethnicity and parent education as covariates; longitudinal models further adjusted for IE in adolescence. IE scores at both time points and regression residuals across all models were normally distributed. We ran three separate cross-sectional models to examine how the following three measures of FI related to IE: (1) household FI in adolescence, (2) adolescent experience of FI and (3) emerging adult experience of FI. The cross-sectional association between household FI and IE in emerging adulthood was not modelled, as household FI was determined via parent report and over half of emerging adults did not live at home full time at EAT 2018. We conducted two separate longitudinal models to assess how (1) household FI in adolescence and (2) the adolescent’s experience of FI related to IE in emerging adulthood. Regression results were reported as unstandardised β coefficients, representing the average IE difference between food-secure and food-insecure participants (after accounting for covariates in adjusted models). Estimated marginal means from adjusted models (i.e. model-based estimates that provide the average IE score within FI status, with adjustment for covariates) were also reported.

Finally, to test whether IE scores in emerging adulthood differed across food security persistence from adolescence to emerging adulthood (using the four-level FI persistence variable described above), we conducted a linear regression model adjusted for sex, age, race/ethnicity, parent education and IE in adolescence followed by pairwise comparisons of estimated marginal means to evaluate the nature of any significant differences.

Given established differences in eating behaviours by gender^(14)^, we also evaluated whether there were significant gender differences in the association between FI and IE by including an interaction term between gender and FI across all models. Because the interaction was not significant in any model, we chose not to present those analyses for parsimony.

We calculated *f* ^2^ to estimate the magnitude of the effect of FI on IE. For adjusted models, *f* ^2^ represents the additional variance in IE that is accounted for when FI is added to the model (above and beyond covariates). Cohen’s conventions for interpreting *f* ^2^ are 0·02 = small, 0·15 = medium and 0·35 = large^(43)^. Cohen’s *d* is used as the effect size measure for pairwise comparisons of IE scores across food security persistence (0·20 = small, 0·50 = medium and 0·80 = large)^(43)^.

## Results

IE significantly decreased from 2010 (*M* = 2·93) to 2018 (*M* = 2·87) in the overall sample, *t*(2751) = 3·03, *P* < 0·01. In adolescence, 41·9 % of participants lived in households with past-year FI per parent/guardian report. The proportion of participants experiencing FI themselves was 14·2 % in adolescence and 22·3 % in emerging adulthood. From adolescence to emerging adulthood, 5·2 % remained food-insecure, 17·0 % became food-insecure, 8·9 % became food-secure and 68·9 % remained food-secure.

### Cross-sectional associations

#### Adolescence

In the unadjusted model (Table 1), average IE scores among adolescents in food-insecure households were 0·08 lower than those in food-secure households (*P* < 0·02). However, when accounting for parent education, sex, race and age, IE scores were not significantly different between adolescents in food-insecure and food-secure households (*P* = 0·09).

**Table 1:** Cross-sectional and longitudinal associations between food insecurity and intuitive eating

Analysis	Food insecurity measure	Unadjusted					Adjusted				
		*b*	se	*t*	*P*	*f* ^2^	*B*	se	*t*	*P*	*f* ^2^
Cross-sectional											
Adolescence	Household	−0·08	0·03	2·41	0·02	0·004	−0·06	0·03	1·70	0·09	0·003
	Adolescent experience	−0·14	0·05	3·08	0·002	0·01	−0·10	0·05	2·27	0·02	0·01
Emerging adulthood	Emerging adult experience	−0·12	0·04	3·19	0·001	0·01	−0·13	0·04	3·43	< 0·001	0·01
Longitudinal											
Adolescence to emerging adulthood	Household	−0·11	0·03	3·33	< 0·001	0·01	−0·08	0·03	2·45	0·01	0·01
	Adolescent experience	−0·09	0·05	1·94	0·045	0·002	−0·04	0·05	0·87	0·38	0·001

IE, intuitive eating.

Adjusted models included parent education, race/ethnicity, sex and age as covariates; longitudinal models were additionally adjusted for baseline IE. Food-secure participants were the reference group across analyses. The unstandardised β coefficient represents the average IE difference between food-secure and food-insecure participants (after accounting for covariates in adjusted models). For example, average IE scores, which range from 1 to 4, were 0·10 lower in adolescents experiencing food insecurity than adolescents who were food-secure. Effect sizes are presented for the individual FI variable (*v*. the entire model); Cohen’s conventions for interpreting *f* ^2^ effect sizes are 0·02 = small, 0·15 = medium and 0·35 = large^(43)^.

Adolescents who reported experiencing FI themselves had IE scores that were 0·10 lower than adolescents who were food-secure in adjusted models (*P* = 0·02; unadjusted: *P* = 0·002).

#### Emerging adulthood

**Fig. 1:**
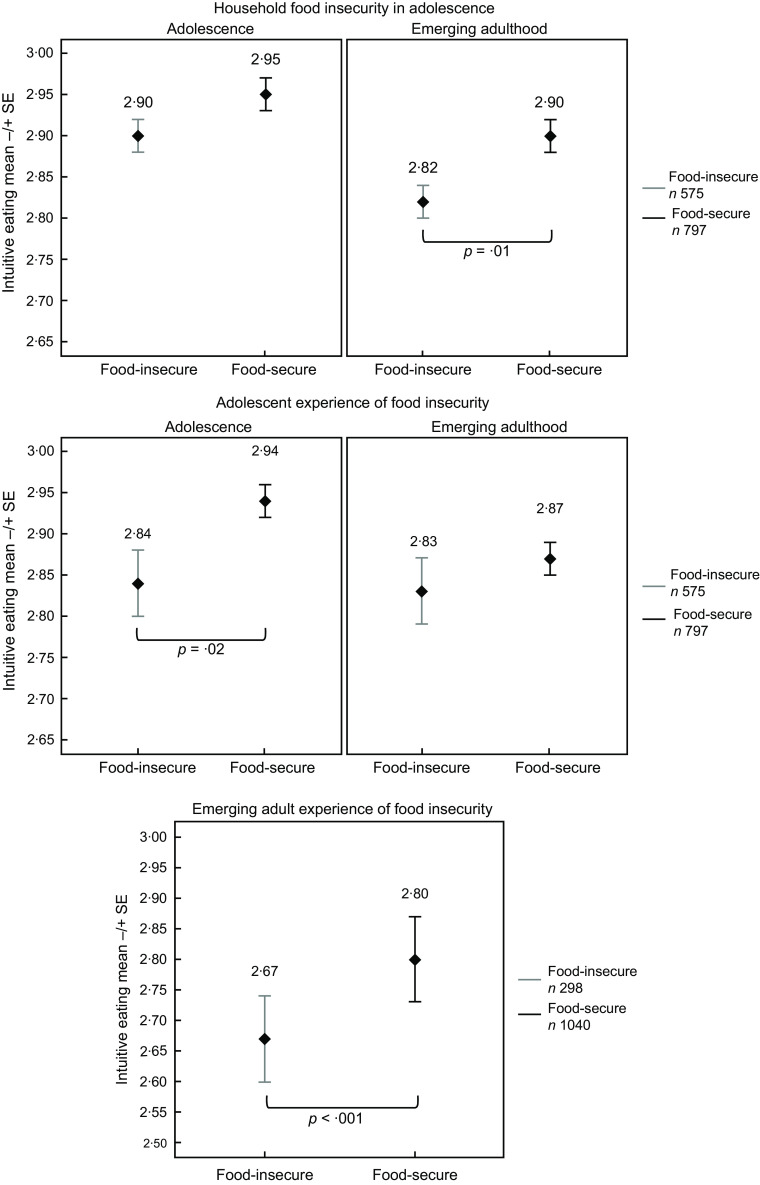
Intuitive Eating Marginal Means by Food Security Status. Note. Means represent the average IE score by food security status and are adjusted for parent education, race/ethnicity, sex, and age; means in emerging adulthood by household food security status are additionally adjusted for adolescent IE. Scores range from 1 to 4 (higher scores reflect greater IE). Statistically significant differences are denoted by brackets.

Emerging adults who experienced FI reported IE scores that were 0·13 lower than those who were food-secure (*P* < 0·001; unadjusted: *P* = 0·001; Fig. 1).

### Longitudinal associations

Among participants who experienced household FI in adolescence, IE scores were 0·08 lower on average in emerging adulthood than participants whose households were food-secure (*P* = 0·01; unadjusted: *P* < 0·001; Fig. 1).

The association between the adolescent’s experience of FI and IE in emerging adulthood was marginally significant in the unadjusted model (*P* = 0·045). However, when accounting for covariates, the association was no longer significant (*P* = 0·38).

### Food insecurity persistence

**Fig. 2:**
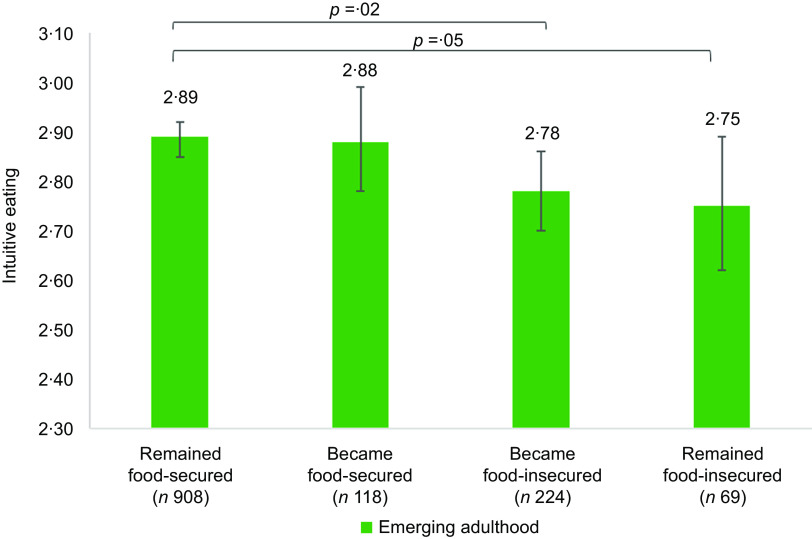
Intuitive Eating in Emerging Adulthood by Food Security Persistence. Note. IE scores adjusted for adolescent IE parent education, race/ethnicity, sex and age. Scores range from 1 to 4. Statistically significant differences are denoted by brackets.

In both the unadjusted and adjusted models, there was a significant association between FI persistence group and IE in emerging adulthood, *F*_unadjusted_ (3, 1324) = 3·42, *P* = 0·02, *f* ^2^ = 0·01, F_adjusted_ (11, 1298) = 8·08, *P* < 0·001, *f* ^2^ = 0·002 (Fig. 2). Specifically, when adjusting for participants’ baseline IE, those who remained food-secure from adolescence to emerging adulthood reported IE scores that were 0·11 higher in emerging adulthood than those who became food-insecure (*P* = 0·02, *d* = 0·14) and 0·13 higher than those who remained food-insecure (*P* = 0·05, *d* = 0·22). Conversely, IE scores in emerging adulthood were nearly identical for participants who remained food-secure (marginal *M* = 2·89) and those who became food-secure (marginal *M* = 2·88; *P* = 0·99). Thus, although adolescents who experienced FI reported lower IE than adolescents who were food-secure, those who became food-secure in emerging adulthood had comparable IE scores to those who were food-secure at both time points.

## Discussion

The purpose of the current study was to investigate: (1) cross-sectional and longitudinal associations between FI and IE in adolescence and emerging adulthood; and (2) whether FI persistence was associated with differences in IE in emerging adulthood. We found partial support for our hypotheses; FI and its persistence were associated with lower IE in cross-sectional and longitudinal analyses, with differences by FI measure (i.e. household *v*. young person-reported). We expected youth-reported FI would be more strongly associated with IE than household FI, but this hypothesis was supported in cross-sectional analyses only. Effect sizes were generally small across analyses. Results suggest that disrupted intake due to FI likely makes eating intuitively more challenging in the short term, whereas household FI may impede IE development over time. Therefore, results lend tentative support to mainstream concerns that IE currently may not be equitably accessible to all^(20,21)^.

In cross-sectional analyses accounting for sociodemographic factors, household FI in adolescence was not significantly associated with IE, whereas adolescent experience of FI *was* related to lower IE. Items on the short, six-item household FI measure that assess hunger and reduced food intake specifically ask about the experiences of the parent or other adults in the home, but not about the children’s experiences. Conversely, the youth measure specifically asks if the *youth* has eaten less than they should or experienced hunger in the prior year. Thus, the lack of a cross-sectional association between household FI and IE in adolescence might be partially explained by research showing that parents in food-insecure households often try to shield their children from the effects of FI, such as forgoing or restricting their own meals so their children can eat^(29)^. Consistent with prior data^(28)^, household FI was more common than adolescent-reported FI, suggesting the intake of adolescents in some food-insecure households may not have been affected. These results suggest the direct experience of hunger or altered intake might be a more salient proximal influence on IE than household-level FI. For someone with current or recent experiences of hunger due to FI, it would be understandable – —even sensible – to take advantage of food availability by eating as much as possible, potentially bypassing feelings of fullness. Moreover, research shows that even acute dietary restriction can disrupt awareness of interoceptive cues such as satiety^(44)^. Thus, youth who have experienced hunger might be less able to detect their internal cues.

Longitudinal analyses displayed the inverse pattern of results, with household FI in adolescence, but not the adolescent experience of FI, predicting lower IE scores in emerging adulthood. It appears that household FI might be a more distal, rather than proximal, influence on IE. The household food security measure was more comprehensive than the youth version, assessing additional factors such as being able to afford to eat balanced meals. Given that household FI is related to many other structural barriers and inequities such as income, employment, healthcare access, housing, neighbourhood safety, discrimination and more^(24,45)^, it could be this multidimensional measure captures the long-term effect of broader inequities that extend beyond experiences of hunger. Because parental modelling is strongly associated with the eating patterns young people develop^(46)^, it is also possible that parents in food-insecure households were less able to model IE for their children.

Young people who remained food-secure across adolescence and emerging adulthood reported significantly higher IE scores in emerging adulthood than participants who became or remained food-insecure from adolescence to adulthood. These results suggest that experiencing FI during the transition to adulthood might be detrimental to the ability to eat according to one’s internal cues. We did not find evidence that persistent FI was uniquely related to IE in emerging adulthood. However, because our measurements were spaced 8 years apart, we were only able to approximate persistent FI and there may have been considerable heterogeneity within groups. To gain a more nuanced understanding of the cumulative effects of persistent FI on IE over time, future research should consider more comprehensive assessments of FI at closer intervals. We also found that IE decreased over time for most participants. The reason for this finding is unclear, as there is no known prior research on the stability of IE from adolescence to emerging adulthood outside of this cohort^(16)^. However, it is possible that heightened sociocultural appearance pressures and engagement with appearance-focused social media in emerging adulthood might make emerging adults more susceptible to using weight or shape control behaviours rather than eating according to their hunger and fullness^(47)^. It is also possible that emerging adulthood is a uniquely challenging time to eat intuitively, as young people may experience instability in schedules and responsibilities as they transition to independence^(48)^. Nevertheless, because youth who experienced persistent food security demonstrated the highest IE scores across time, maintaining consistent access to food might help buffer decrements to IE during the transition to adulthood.

Taken together, it appears that FI may exert both immediate and potentially long-term effects on IE. In particular, it appears acute experiences of hunger related to inadequate or intermittent food availability likely make IE particularly challenging in both adolescence and adulthood. Long-term, household FI may negatively affect IE development, potentially as one component of broader systemic inequities perpetuating nutritional and health disparities. Further research is needed to validate these findings, as effects were generally small, suggesting FI is likely one of many factors (e.g. sports participation, discrimination and mental health) affecting IE across adolescence and emerging adulthood. Nevertheless, it is possible even small changes in IE over the critical developmental periods of adolescence and emerging adulthood could have cumulative effects throughout the life course^(49)^.

Although results lend tentative support to mainstream concerns that IE is a privileged approach, the problem is not necessarily IE itself, but the inequitable allocation of resources and disproportionate structural barriers that likely make IE inaccessible for many. The core framework of IE emphasises flexibility and attunement to one’s own needs^(13)^. For those experiencing inconsistent food availability or access only to processed, packaged and convenience foods, IE would encourage meeting one’s needs by eating the food that is accessible in the quantity needed^(13)^. Attempting to restrict intake and adhere strictly to hunger and satiety cues when food access may become restricted again would not align with meeting one’s needs, nor would choosing to not eat less nutritionally dense foods when they are what are accessible. However, continued focus and investment within research and policy are needed to achieve health equity so that individuals themselves do not bear the primary responsibility for surmounting the structural barriers they face. Policies that promote greater access to nutrient-dense foods might aid IE in individuals experiencing FI. However, improving economic conditions so that fewer individuals are affected by issues such as FI, which contribute to and widen health disparities, is a crucial long-term goal^(3,4,45)^.

### Strengths and limitations

Strengths of this study include that it is the first known investigation of both cross-sectional and longitudinal associations between FI and IE from adolescence to emerging adulthood. Moreover, this study was conducted in a socio-economically, ethnically and racially diverse population-based cohort, which is a considerable strength given most IE research has been conducted with majority White, female and/or undergraduate samples^(50)^. Similarly, the majority of IE research is cross-sectional^(50)^ and examines IE as a predictor rather than an outcome. Elucidating factors that facilitate or hinder IE development and maintenance will be critical to designing interventions that help foster this adaptive eating style among young people.

It is also important that we acknowledge this study’s limitations. Although the longitudinal design is a strength, it is likely factors contributing to lower IE among individuals experiencing FI are complex and might onset prior to adolescence. For instance, we were unable to examine potential mechanisms contributing to lower IE among individuals with FI, such as the role of neurobiology or altered interoceptive awareness. Moreover, individuals experiencing FI likely face other structural inequities that could compound effects on eating behaviours (e.g. limited availability of nutrient-dense, satiating foods). Additionally, FI has psychosocial dimensions (e.g. worry, stigma) not considered in the present analyses, but that are likely relevant to IE. For instance, experiencing stigma when utilising food assistance programmes can prevent individuals from accessing these crucial services, thereby potentially intensifying FI^(51)^. Further, the mental health consequences associated with FI (e.g. stress, worry)^(3)^ could impede IE by disrupting attunement to internal cues and driving emotional eating. Therefore, future research should examine the associations between FI’s psychosocial dimensions and IE. Another limitation is that FI was assessed for the prior year only at two time points spaced 8 years apart. Thus, we were only able to approximate chronic FI, and there may have been considerable heterogeneity in the pattern and persistence of FI within groups. Future research should consider more comprehensive assessments of FI at closer intervals to evaluate the cumulative effects of persistent FI on IE over time. Finally, to ease participant burden, we used a brief, three-item measure of IE that does not capture the breadth of the multidimensional IE construct. It would be interesting for future work to assess whether associations between FI and IE differ across IE’s domains using the full IES-2 scale^(52)^.

## Conclusion

In conclusion, the current study found that youth who experienced past-year FI had lower IE in both adolescence and emerging adulthood. Household FI in adolescence appeared to be a distal, rather than proximal, risk factor for lower IE in emerging adulthood. Youth who experienced persistent FI had lower IE scores in emerging adulthood than those who remained food-secure. Taken together, results suggest FI might be yet another structural barrier economically disadvantaged youth face to IE and associated improvements in health outcomes. Further research is needed to clarify how sociocultural factors (and their intersections) affect the development and stability of IE over time to inform effective prevention and treatment efforts. Because poverty and FI are key social determinants of health^(45)^, successful prevention and treatment interventions must also consider and seek to modify the structural and systemic barriers to health-promoting eating behaviours that ultimately perpetuate health disparities. Future research should consider strengthening individual- and group-level IE interventions by taking a multi-level approach, working to both improve food security and help young people eat according to hunger and satiety cues.
