# Is intuitive eating a privileged approach? Cross-sectional and longitudinal associations between food insecurity and intuitive eating – CORRIGENDUM

**DOI:** 10.1017/S1368980023001027

**Published:** 2023-08

**Authors:** C Blair Burnette, Vivienne M Hazzard, Nicole Larson, Samantha L Hahn, Marla E Eisenberg, Dianne Neumark-Sztainer

The authors would like to apologise for an error in the above article. During submission Samantha L Hahn’s name was given as Samantha A Hahn.

This has been updated in the original article.
